# Relationships between and formation dynamics of the microbiota of consumers, producers, and the environment in an abalone aquatic system

**DOI:** 10.1371/journal.pone.0182590

**Published:** 2017-08-07

**Authors:** Jing-Zhe Jiang, Wang Zhao, Guang-Feng Liu, Jiang-Yong Wang

**Affiliations:** 1 Key Laboratory of Aquatic Product Processing, Ministry of Agriculture, South China Sea Fisheries Research Institute, Chinese Academy of Fishery Sciences, Guangzhou, China; 2 Tropical Fisheries Research and Development Center, South China Sea Fisheries Research Institute, Chinese Academy of Fishery Sciences, Sanya, China; University of Waikato, NEW ZEALAND

## Abstract

An ecosystem is a community comprising living and nonliving components of the environment. Microbes are ubiquitous elements in each of these components. The dynamics of microbiota formation in an ecosystem is important to elucidate, because how the different components of a system exchange microbes, and how the microbes control ecological processes remain unresolved. In this study, an abalone, *Haliotis diversicolor*, seed-nursing pond was used as a model system. We first examined changes in bacterial communities during the seedling cultivation of this herbivorous juvenile aquatic invertebrate animal. Denaturing gradient gel electrophoresis (DGGE) and pyrosequencing were used to analyze bacterial community dynamics and spatio-temporal interactions of different system components: consumers (abalone), producers (algae or a substrate), and the environment (water). DGGE fingerprints revealed that the developmental stages of abalone influences bacterial communities of both the abalone and substrate. Although the communities in water fluctuated daily, they could be divided into two clusters that coincided with abalone stages, reflecting the transition from larva to juvenile at around day 21. Pyrosequencing showed that the microbiota in the abalone and substrate had more operational taxonomic units in common than that of either with water. The Bray-Curtis similarity index was used to quantify the formation dynamics of microbiota among the various components of the system. The bacterial communities in producers and consumers showed similar changes. These communities were unstable at the beginning and then slowly stabilized over time. The environmental bacterial community was more stable than the bacterial communities in consumers and producers, and may have been the basis for stability in the system. Our research provides insights into the dynamics of microbiota formation in various biotic elements of a system that will contribute to predictive systems modeling.

## Introduction

An ecosystem is an interactive community composed of living organisms (plants, animals, and microbes) and nonliving components of their environment (e.g., air, water, and mineral soil, etc.). The complex network of interactions between organisms and between organisms and their environment are the core concern in ecological studies. A network is controlled by external and internal factors. External factors such as climate, the nature of the soil, and topography control the overall structure and function of an ecosystem. In contrast, internal factors such as species, competition, disturbances, succession, and decomposition not only control ecosystem processes, but also are controlled by these processes [1].

An abalone seed-nursing pond is a simple aquatic system composed of three major constituents: abalone, water, and an adherent substrate (plastic films with algae attached). In this system, abalones are consumers living on the substrate and feeding on the algae attached to the film. Water serves as the environment for the abalones and algae. The plastic film provides the adhesive substrate for algae and accumulates the excrement of the consumers [2]. As the system is established, abalone seedlings hatch, and the adhesive substrate is dominated by pre-cultivated diatoms. Through time, abalone excrement gradually accumulates. Therefore, adhered material is a mixture consisting mainly of abalone food at an early stage of abalone development that slowly transitions to accumulated excrement at a later stage [3]. Because of the simplicity of many aquacultures, they are good models to study the relationships between different microbial communities in a system.

Although the three components of our selected system are very different from each other, they have a common link: the microbes living in or on them. Microorganisms, the backbone of all ecosystems, are ubiquitous. They play a variety of essential functions in each of the components of the system. For example, water microbes can be decomposers, producers (e.g., cyanobacteria), or promoters of elemental cycling (e.g., carbon or nitrogen fixation). Their presence promotes regular energy flow and cycling of nutrients in the system, improving the efficiency of resource usage [4]. All man-made structures immersed in a marine environment are quickly colonized by a variety of micro- and macroorganisms [5]. On plastic films, bacteria establish close relationships with their neighbors in the phycosphere, a zone surrounding the algae [6]. The interactions between algae and bacteria not only dynamically control the growth of other bacteria [3,7], they also influence the settlement of marine invertebrate larvae [8]. The population of microorganisms living in an animal body is called the microbiome or microbiota [9]. The various functions of the gut microbiome of humans and other vertebrates, including barrier functions, metabolic reactions, trophic effects, and maturation of a host’s innate and adaptive immune responses, have been analyzed [10].

Although the roles of microbes in certain hosts or environmental niches have been extensively studied, their roles and interactions in the system as a whole are largely unknown. Revealing the factors and processes that shape the dynamics of host-associated microbiota under natural conditions is important to understanding and predicting an organism’s response to a changing environment [11]. As ubiquitous elements of both living and nonliving components of an ecosystem, microbes are actively exchanged among parts of the ecosystem. Therefore, microbes are most likely acting as internal factors that not only affect processes in the ecosystem, but also provide stability between components of the system. However, how microbes are exchanged between components of a system and, specifically, how producers, consumers, and the environment influence each other’s microbial communities is still unknown. Using denaturing gradient gel electrophoresis (DGGE) and high-throughput sequencing, the composition of a bacterial community can be determined based on 16S rRNA sequencing. In this study, we collected time-series samples from three components of an abalone seed-nursing pond system. Profiling of the 16S rRNA was used to characterize bacterial communities. The Bray-Curtis (BC) similarity index was used to quantify the dynamics of microbiota formation in different organisms and the environment, thus defining their relationships system-wide.

## Materials and methods

### Sample collection

Abalone (*Haliotis diversicolor* Reeve, 1846) hatching was performed following the guidelines outlined by You *et al*. [12] and Zhang *et al*. [2]. Three identical nursing ponds, 7 m × 3 m × 1 m in size and with ~16 t of filtrated seawater, were used in this study. Polythene films were added as a substrate for abalone larva and diatom attachment. Throughout the study period (from day 0 to 41), water temperature, salinity, light intensity, and initial larval density were maintained identically for all ponds. At the beginning, diatoms were pre-cultivated for 3 days in static water before the larvae were added on day 0. The free-swimming larvae were lecithotrophic on days 1 and 2 and were collected with a 100-μm mesh screen. The larvae started settling on the films and fed on the attached algae at approximately day 3. On day 4, the running water was introduced into the ponds, with two complete water exchanges per day. Because the settled larvae could not be visibly distinguished from diatoms from day 3 to day 5, visible larvae or early juveniles were carefully removed from the films with a brush beginning at day 6. For substrate samples, three 100 × 100-mm films were excised at three different locations in each pond every day. The above samples were stored at −80°C after they were rinsed in sterile seawater. One-liter water samples from each pond were filtered through 0.22-μm nitrocellulose membrane filters every day, and filters were stored at −80°C.

### DNA extraction and PCR-DGGE

We combined triplicate samples for DNA extraction to minimize random errors resulting from sample differences. Abalone DNA was extracted using the E.Z.N.A. Bacterial DNA Kit (Omega, Norcross, GA, USA) following the centrifugation protocol with minor modifications. Abalone tissues (50–100 mg) were firstly homogenized in 100 μL TE buffer and 10 μL lysozyme, and incubated at 37°C for 10 minutes. Then following operations are carried out according to the instruction. The Omega E.Z.N.A. Water DNA Kit was used to extract bacterial DNA from film and water samples. Fragments of 16S rDNA for the DGGE analysis were amplified using a nested PCR method with first-round primers 8F/1492R (8F: 5′-AGAGTTTGATCCTGGCTCAG-3′; 1492R: 5′-GGTTACCTTGTTACGACTT-3′) [13] and second-round primers 341F-GC/534R for the V3 hyper-variable regions (341F-GC: 5′-CGCCCGCCGCGCGCGGCGGGCGGGGCGGGGGCACGGGGGGCCTAGGGGAGGCAGCAG-3′, 534R: 5′-ATTACCGCGGCTGCTGG-3′) [14]. PCR was performed as described previously [3]. Amplified products were analyzed by DGGE using a BioRad DCode Universal Mutation Detection System (BioRad, Hercules, CA, USA) and applied to 8% w/v polyacrylamide gels with denaturing gradients that ranged from 55 to 75% with 1× TAE running buffer, and that were run at 60°C for 11 h at 100 V. Gels were then stained with 1× SYBR Gold Nucleic Acid Gel Stain (Life Technologies, Carlsbad, CA, USA) for 30 min. Photos were taken with a Tanon-2500 (Shanghai, China).

### High-throughput sequencing of the 16S rDNA

PCR amplification, purification, pooling, and pyrosequencing were performed following the procedure described by Liu *et al*. [15], with minor modifications. The V1–V3 hyper-variable region of the bacterial 16S rDNA was amplified with primers B-27F and A-533R (454 Life Sciences, Branford, CT, USA). Sequencing starts from A-533R to B-27F (A and B represent the 454 adaptors, where A contains the index sequences). PCR was carried out in triplicate 20-μL reactions containing 100 nM of each primer, 10 ng of template, 0.25 mM dNTPs, and 1 U polymerase (TransStart-FastPfu DNA Polymerase, TransGen Biotech, Beijing, China). The following thermal program was used for amplification: 95°C for 2 min, followed by 25 cycles of 95°C for 30 s, 55°C for 30 s, and 72°C for 30 s, and then an extension at 72°C for 5 min. The PCR products were pooled and purified. Equal amounts of the products from each sample were combined in a single tube to be run from the A-end on a Roche Genome Sequencer GS-FLX Titanium platform at Majorbio Bio-Pharm Technology Co., Ltd. (Shanghai, China).

### Data processing and statistical analysis

The PCR-DGGE band signals were digitized using Quantity One software (Bio-Rad). The Shannon-Wiener index of diversity (H) [16] was used to determine the diversity of taxa present in different components of the system. A principal component analysis (PCA) was conducted using Canoco for Windows 4.5 (Wageningen, Netherlands). A similarity matrix was constructed by applying Dice's similarity coefficient in SPSS software. Phylogenetic trees were constructed using the unweighted pair group method with arithmetic averages (UPGMA).

For the pyrosequencing data, only reads longer than 200 base pairs (bp), with an average quality score greater than 25, and without any ambiguous base calls were included in the subsequent analyses. The trimmed and unique sequences were used to determine operational taxonomic units (OTUs) at the 97% similarity level. Both the rare OTUs with only 1 or 2 mapped reads and chimeric OTUs were discarded. The remaining OTUs were then subjected to a BLAST search against the SILVA database (Version 111), and only prokaryotic sequences were kept for further analysis. Relationships and distances between all of the sequenced samples in the community are shown in the phylogenetic tree (Jaccard index) and PCA. The statistical distance between each pair of samples was determined using the weighted UniFrac metric [17]. Except for the PCA, the above analyses were performed in Mothur [18].

**Fig 1:**
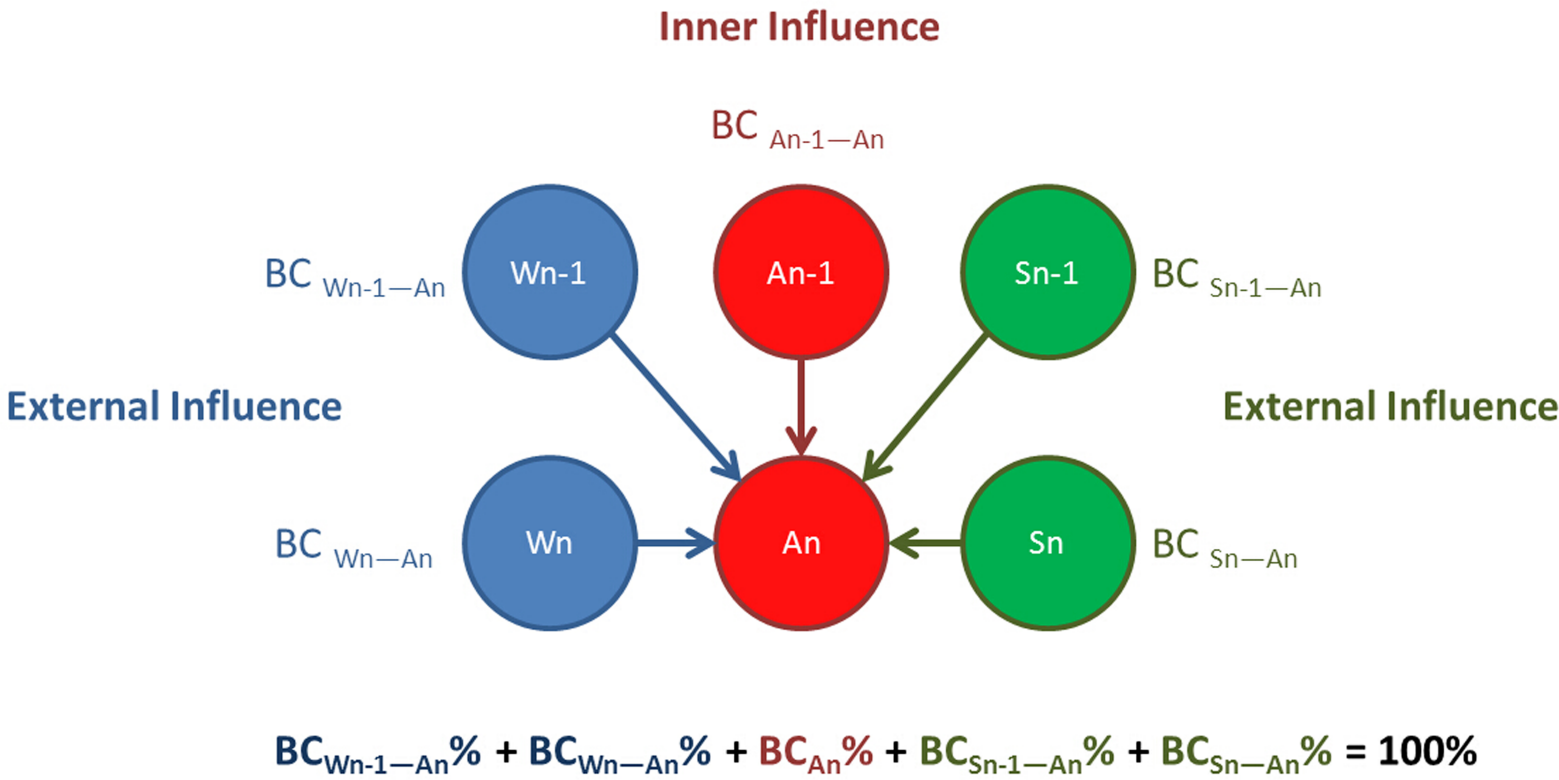
Schematic representation of the BC% analysis of the abalone seed-nursing ecosystem. “A,” “S,” and “W” represent different components of the ecosystem with direct microbial exchanges. Comparisons of bacterial communities were only conducted for two samples that were spatially or temporally adjacent, e.g., An-1&An, Wn-1&An, Sn&An. The microbiome of “An” is mainly influenced by five spatially and temporally adjacent samples: Wn-1, An-1, Sn-1, Wn, and Sn. The influence of “W” or “S” was external and that of “An-1” was internal. When the BC values of “An” are considered 100%, the BC percentage (BC%) reflects the strength of the influence on “An”. BC, Bray-Curtis similarity value; A, Abalone; S, Adherent substrate; W, Water; n − 1, Time point prior to “n.”

A methodology, based on BC similarities, was used for the analysis of spatio-temporal interactions between bacterial communities associated with different components of the system. BC similarity is a statistic used to quantify the compositional similarity between two different communities, based on counts of common species. The index of similarity is:

$${BC}_{ij}=\frac{2C_{ij}}{S_{i}+S_{j}}$$

where *C*_*ij*_ is the sum of the lesser values for only those species (OTUs) in common between two samples, and *S*_*i*_ and *S*_*j*_ are the total number of species (OTUs) in each sample [19]. First, two temporally close samples are defined as “n-1” and “n,” with “n-1” referring to the time point before “n.” Taking abalone sample “A” as an example (Fig 1), the microbiome of “An” is influenced mainly by five spatially and temporally adjacent samples and include spatially close samples (“Sn” for substrate and “Wn” for water), temporally close samples (An-1), and spatio-temporally close samples (Sn-1 and Wn-1). The BC similarity values between “Sn” and “An” were termed BC_Sn-An_; the BC value between “An-1” and “An” is named BC_An-1-An_, and so on. If the five spatially and temporally adjacent BC values together are considered 100%, the percentage of BC_An-1-An_ (BC_An-1-An_%) would reflect the community stability (inner influence) of “A,” whereas the values BC_Sn-An_% and BC_Sn-1-An_% would reflect the external influences of the “S” community on “A,” and BC_Wn-An_% and BC_Wn-1-An_% would show the external influences of “W” on “A.” To exclude the effects of different the sizes of sample datasets, data from the above analyses were normalized, and data were processed in Excel.

## Results

### DGGE analysis of bacterial communities

**Fig 2:**
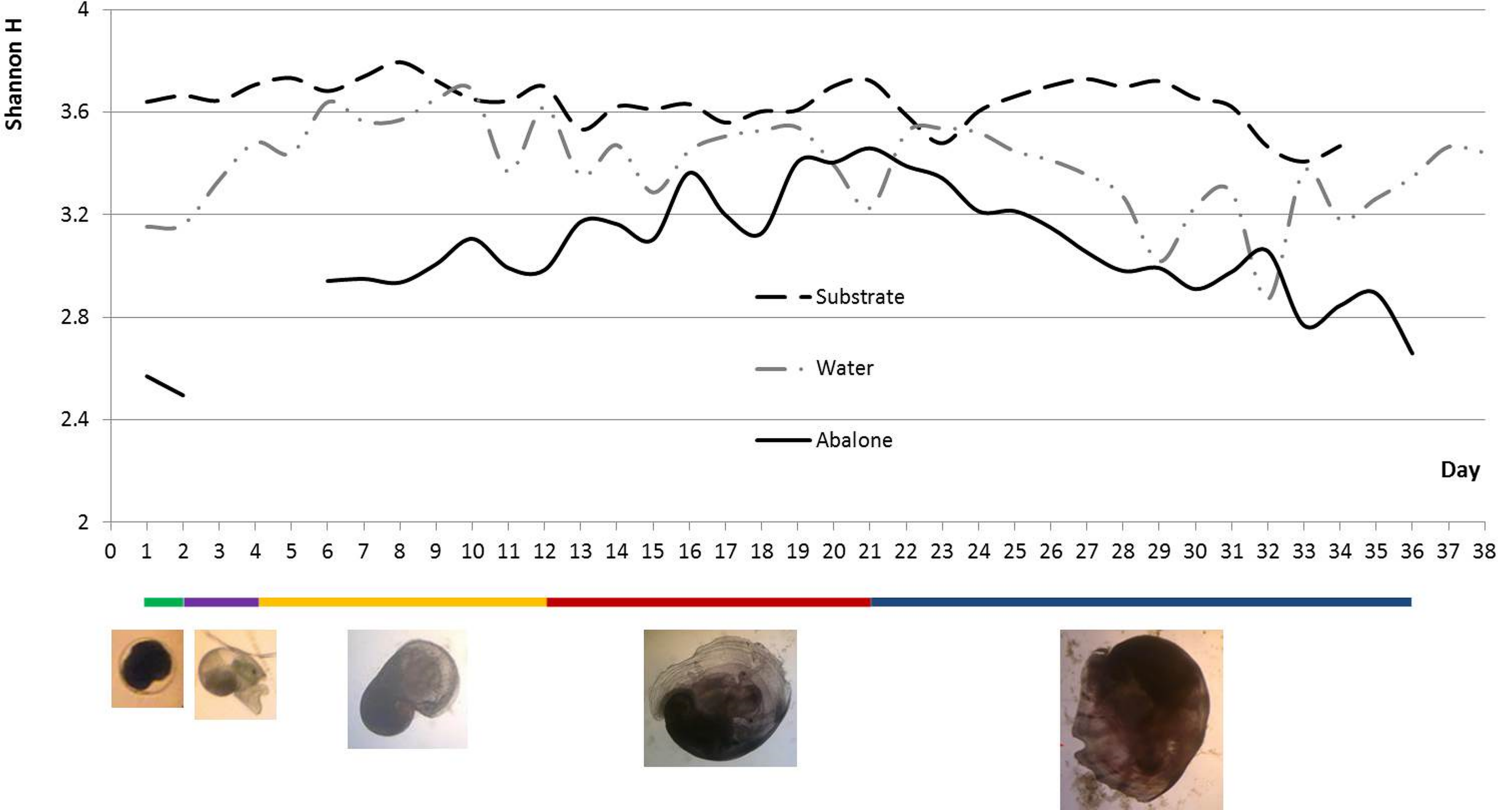
Changes in α-diversity (Shannon-Wiener indexes) of each component in the abalone seed-nursing system over time. Different lengths of colored bars show different developmental stages of the abalone. Green, trochophore stage (0–2 days); Purple, creeping larva stage (2–4 days); Yellow, peristomial shell larva stage (4–12 days); Red, differentiation stage (12–21 days); Blue, juvenile (> 21 days).

**Fig 3:**
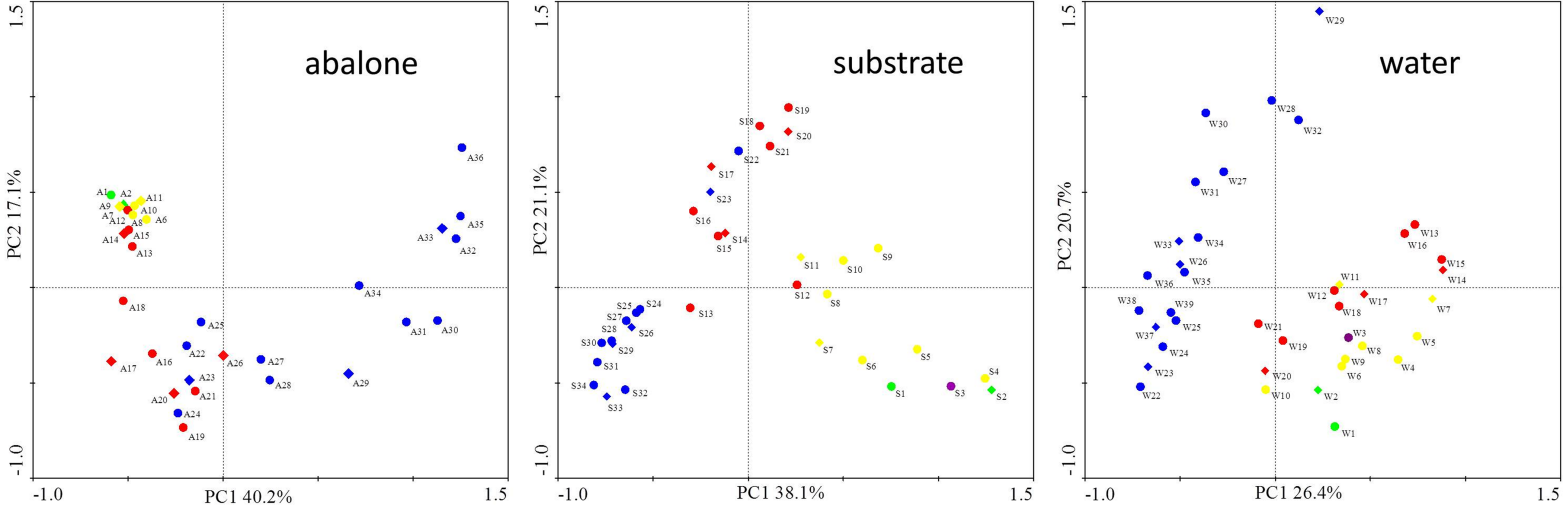
PCA plots based on bacterial denaturing gradient gel electrophoresis patterns of the three components in the abalone seed-nursing system. Different colored dots indicate the different developmental stages of the abalone. Green, trochophore stage (0–2 days); Purple, creeping larva stage (2–4 days); Yellow, peristomial shell larva stage (4–12 days); Red, differentiation stage (12–21 days); Blue, juvenile (> 21 days).

DGGE of each sample yielded hundreds of PCR bands (S1 Fig); that is to say, the samples were appropriately prepared. Changes in the α-diversity (Shannon-Wiener indices) of each component over time were plotted (Fig 2). Generally, bacteria in the substrate sample had the highest diversity, followed by water, and then the lowest in abalone. It is worth mentioning that from day 1 to day 21, the abalone bacterial indices showed an upward trend, followed by a downward trend. The PCA analysis based on DGGE patterns revealed that the diversity of the abalone microbiota was closely related to their developmental stages, and the distances between different time points in the same developmental period were relatively close on the PCA map (Fig 3). Interestingly, the substrate was also associated with abalone growth; samples of the substrate that were collected during the same stage clustered together in the PCA analysis. Even the water microbiota formed two clusters—one of samples collected before the 21st day and one of samples collected after, which is when the abalone transitioned from the differentiation stage to the juvenile stage. Three unweighted phylogenetic trees were constructed based on DGGE profiles (S2 Fig). All three samples showed chronological clustering in the trees. However, branching in the trees appeared to have no relationship with abalone developmental stages. Based on the structure of the trees, 11 abalone, 10 substrate, and 11 water samples, collected at the same time points, were selected for pyrosequencing (red branches).

### Pyrosequencing of the bacterial communities

**Fig 4:**
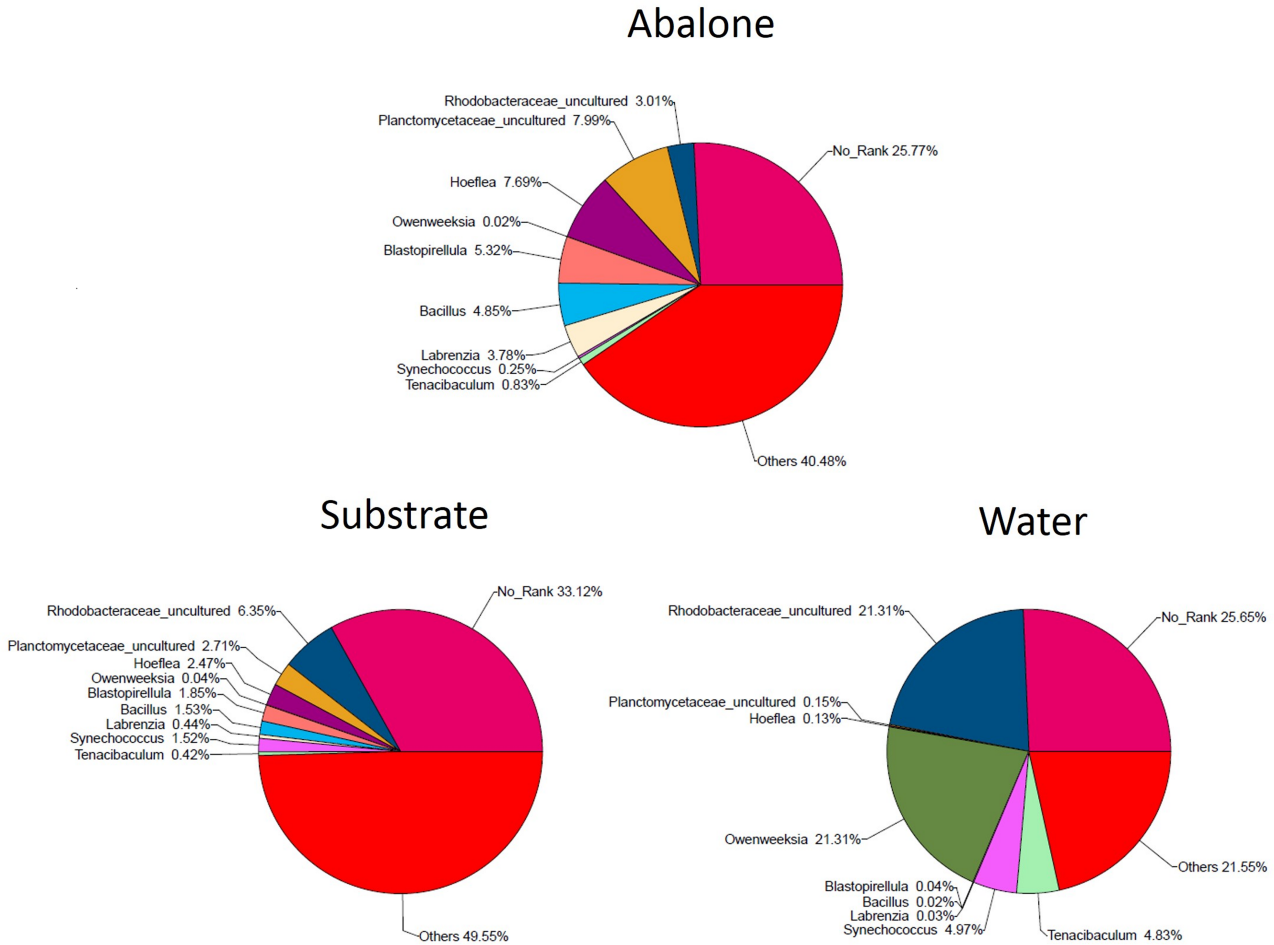
Relative abundance of major bacterial genera recovered from the three components in the abalone seed-nursing system.

The bacterial communities in 32 samples obtained from three components of an abalone seed-nursing system were profiled by pyrosequencing of the 16S rRNA V1–V3 hyper-variable region. In total, 260,586 high-quality reads were generated, with an average length of 455 bp and 8,143 reads per sample (S3 Fig and S1 Table). All sequences were deposited in the GenBank Short Read Archive (SRR1462395). With a similarity threshold of 97%, we identified a total of 23,802 OTU sequences, with 12,096 for the abalone, 11,987 for the substrate, and 5,338 for the water. S4 Fig shows the rarefaction analysis results of all samples. The pyrosequencing results showed that the predominant genus in abalone was *Planctomycetaceae_uncultured*, followed by *Hoeflea*, *Blastopirellula*, and *Bacillus* (Fig 4). This is very similar to the bacterial profiles of the substrate. The above genera also accounted for a large proportion of the bacteria in the substrate; however, *Rhodobacteraceae_uncultured* replaced *Planctomycetaceae_uncultured* as the dominant genus. *Rhodobacteraceae_uncultured* and *Owenweeksia* were most abundant in water, accounting for 40% of the total bacterial community. The other two genera present in water were *Synechococcus* and *Tenacibaculum*, which accounted for less than 10% of the microbiota present (Fig 4). S5 Fig shows the structure of the microbiota in each sample, listed in chronological order. The changes in bacteria community structures in each components of the system were not random, but slowly changed through time.

### Relationships among three bacterial communities

**Fig 5:**
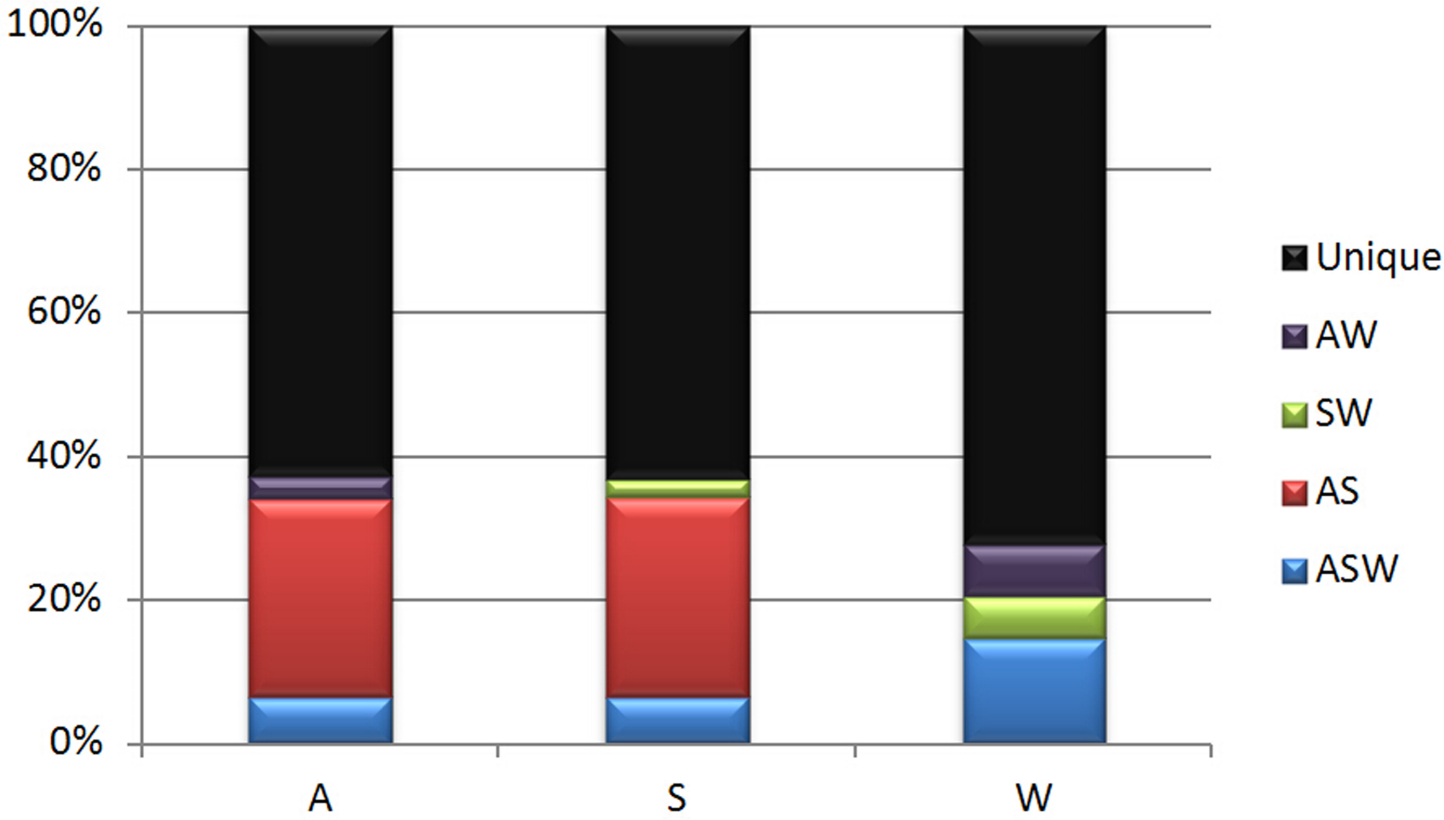
Common operational taxonomic unit (out) percentages of the three components in the abalone seed-nursing ecosystem samples. A, Abalone; S, Adherent substrate; W, Water. Unique, unique operational taxonomic units (OTUs) in each sample; AW, SW, and AS, common OTUs in two samples; ASW, common OTUs in all three samples. The numbers of OTUs in each sample type: A, 119,722; S, 64,431; W, 49,217.

To study the relationships between the three components, three analytical methods were used. First, OTU reads that were shared between components were determined. In total, 783 (out of 23,802) OTUs were present in all of the samples; these were defined as ASW (abalone, substrate, water) OTUs. Abalone and substrate samples (AS) had 4,108 common OTUs in common, whereas substrate and water (SW) shared 1,096 OTUs and abalone and water (AW) shared 1,171 OTUs. To exclude errors because of different sizes of sample datasets, OTU percentages were calculated. AS OTUs accounted for 27.5% and 27.7% of OTUs in abalone and substrate, respectively, which were much higher percentages than the 3.2% and 7.3% AW OTUs in abalone and water, respectively, and 2.6% and 5.9% SW OTUs in substrate and water (Fig 5). Consequently, the percentages of unique OTUs in abalone (62.7%) and the substrate (63.1%) were both lower than that in water (72.2%) because of the higher percentage of AS OTUs.

**Fig 6:**
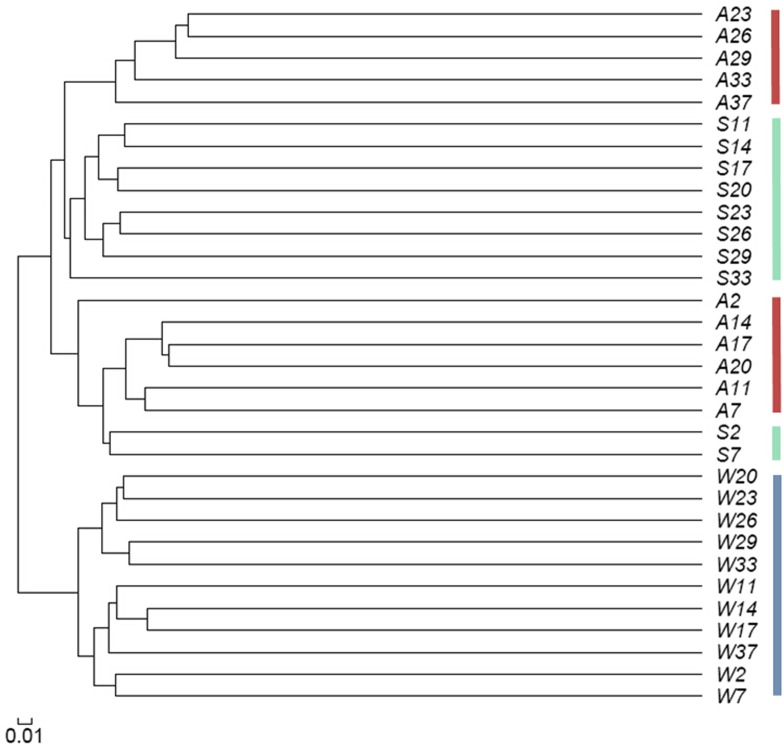
Phylogenetic tree of bacterial communities in abalone seed-nursing ecosystem samples. A, Abalone; S, Adherent substrate; W, Water. Arabic numerals, sampling days. The phylogenetic tree was constructed using Jest (Jaccard coefficient using richness estimators) in Mothur.

A phylogenetic tree of all high-throughput sequencing samples was also generated. Abalone and substrate samples were so similar that they did not segregate in two different branches, but were mixed on one branch (Fig 6). More specifically, the earlier S samples (S2 and S7) clustered with the earlier A samples (A2–A20), and the later S samples (S11–S33) clustered with the later A samples (A23–37). However, the water samples were all on a separate branch, indicating that there were differences between the bacterial communities in water and the other two system components.

**Fig 7:**
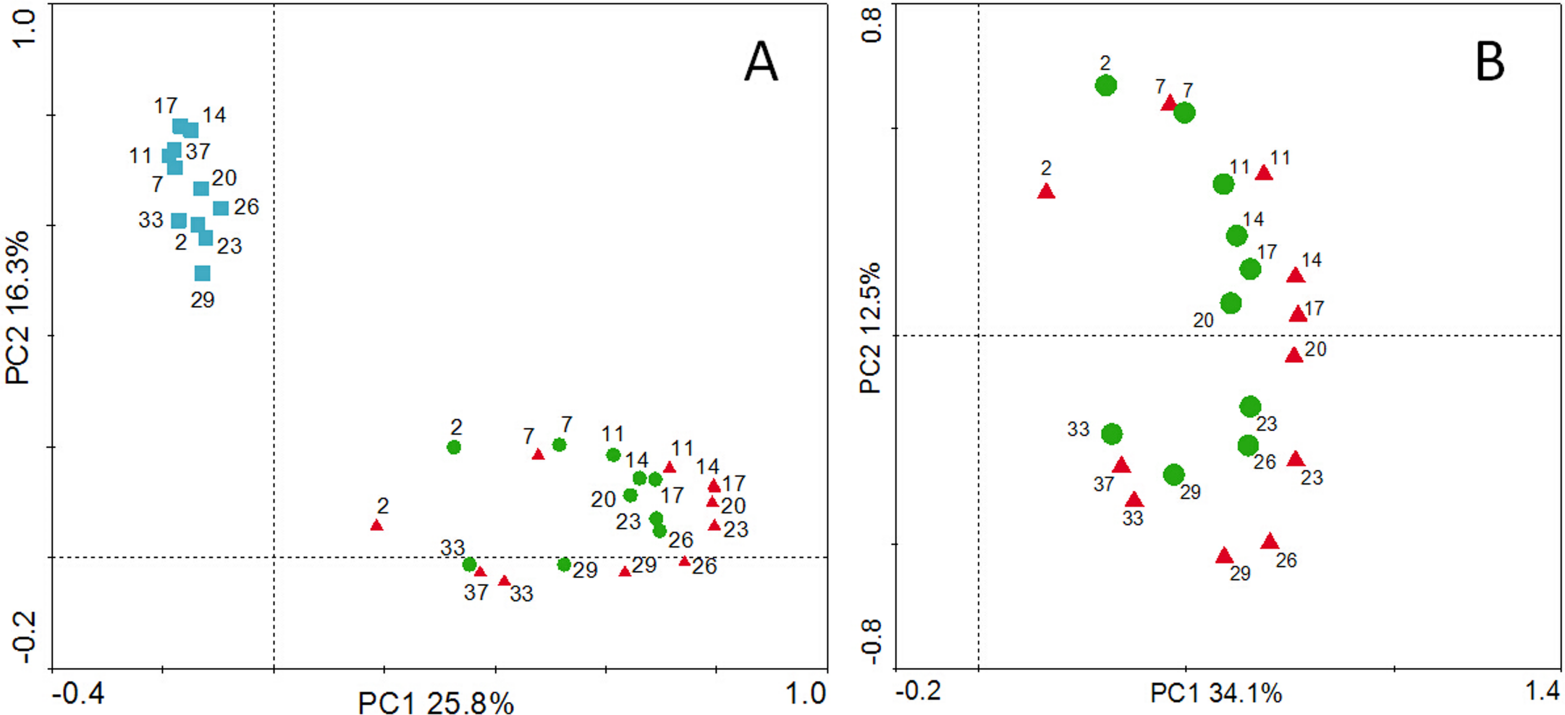
Principal component analysis of bacterial communities in abalone seed-nursing ecosystem samples. A, with water samples; B, without water samples; blue square, water; green circle, adherent substrate; red triangle, abalone; Arabic numerals, sampling days. Results were plotted using Canoco for Windows 4.5, and the 5,000 most abundant operational taxonomic units of the analyzed samples were used in this analysis. Log transformation (Y' = log (Y + 1)).

Finally, PCA was conducted to define the relationships among samples in more detail. In Fig 7A, water samples were shown to be cluster alone, whereas the A samples clustered with the S samples, mirroring with the results of the phylogenetic analysis (Fig 6). When only the AS samples were analyzed, a clearer relationship was observed (Fig 7B). Although the distance between A and S on day 2 was long, A and S were closer together in the days that followed. In conclusion, the above analyses indicated that the consumer and producer had a closer relationship with one another than either with the environment.

### Spatio-temporal interactions among the three bacterial communities

**Fig 8:**
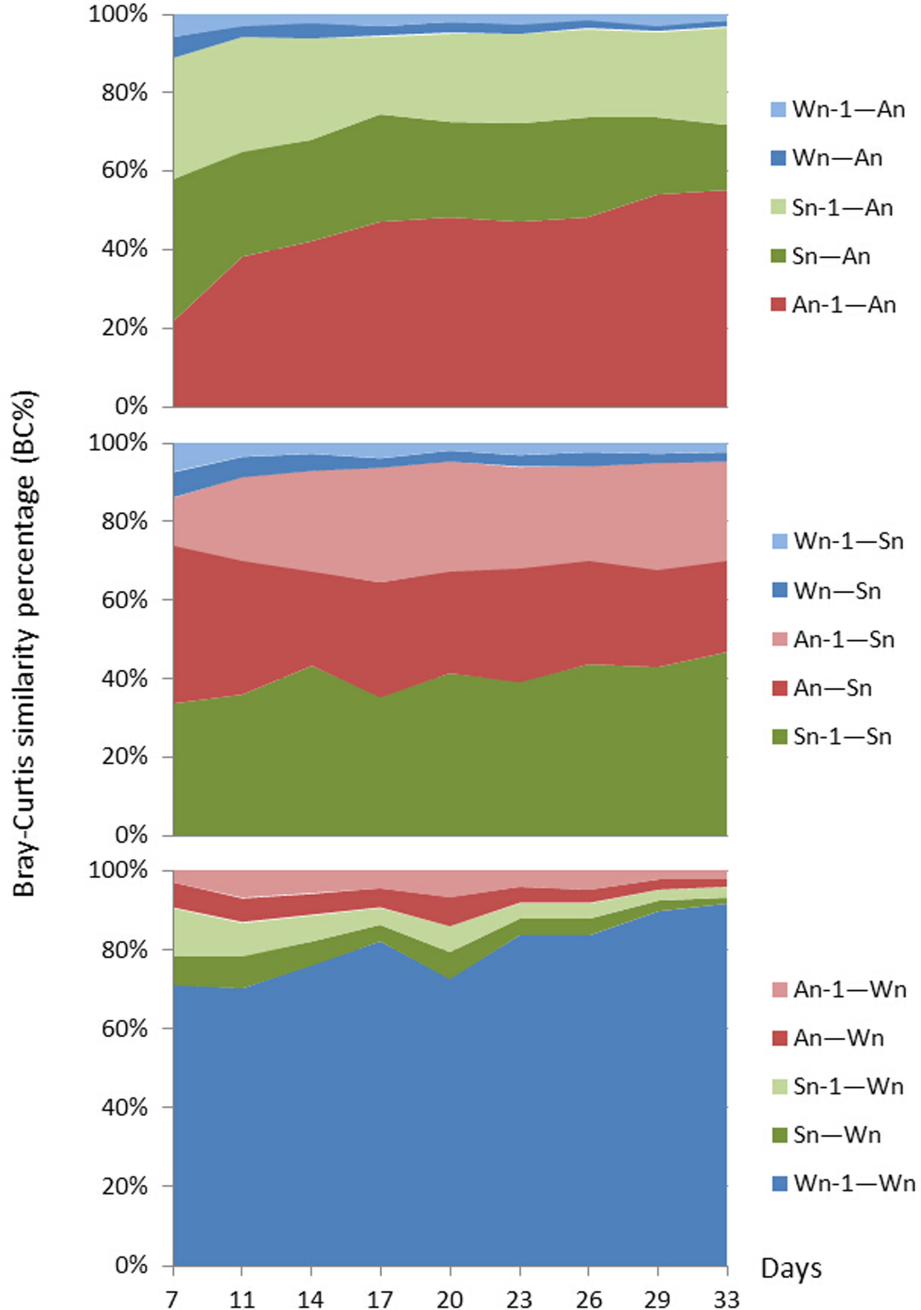
Effects of the three bacterial communities on each other in the abalone seed-nursing ecosystem based on a Bray-Curtis similarity percentage (BC%) analysis. A, Abalone; S, Adherent substrate; W, Water; n − 1, time point prior to “n.” Using the A sample as an example, the microbiome of A is influenced by two spatially (Sn-An and Wn-An), one temporally (An-1-An), and two spatio-temporally (Sn-1-An and Wn-1-An) adjacent samples. Wn-1-An (light blue) indicates the BC_Wn-1-An_ percentage in a total of five BCs of A; Wn-An (dark blue) indicates the BC_Wn-An_; Sn-1-An percentage (light green) indicates the percentage of BC_Sn-1-An_; Sn-An (dark green) indicates the percentage of BC_Sn-An_; and An-1-An (red) indicates the percentage of BC_An-1-An_. This can be repeated for the additional samples. For details, please refer to Fig 1 and Material and Methods.

The spatial and temporal interactions between the three bacterial communities were quantified using the BC% method (Fig 8). For the abalone samples, the inner BC_A_% was only 21.6% at the beginning of the formation of the microbiota, then it gradually rose to 55.1%, indicating that the abalone gut microbiome stability increased as the whole system stabilized. In contrast, external influences, indicated by BC_S-A_% (BC_Sn-An_% + BC_Sn-1-An_%) and BC_W-A_% (BC_Wn-An_% + BC_Wn-1-An_%), both declined from 67.2% to 41.4% and from 11.2% to 3.4% independently, indicating that the effects of the substrate and water on these bacterial communities weakened. As with the abalone, the inner BCs% of the adherent substrate experienced increased slightly from 33.5% to 44.6%, and the external BC_W-S_% declined from 13.7% to 4.5%, from the beginning to the end of the experiment. However, the BC_A—S_% value was relatively stable at around 50% during the whole period. Conversely, the progression of the bacterial community in the water differed from that of the abalone or substrate. First, the inner BC_W_% was much higher than both BC_A_% and BC_S_%, which was 71.0% at the beginning and increased to 91.5% on day 33. This indicated that the bacterial community in the environment was highly stable. Consequently, the external BC_S-W_% and BC_A-W_% values were both low. Whereas BC_S-W_% dropped markedly from day 7 (19.6%) to day 33 (4.3%), BC_A-W_% dropped only slightly from 9.4% to 4.1%.

**Fig 9:**
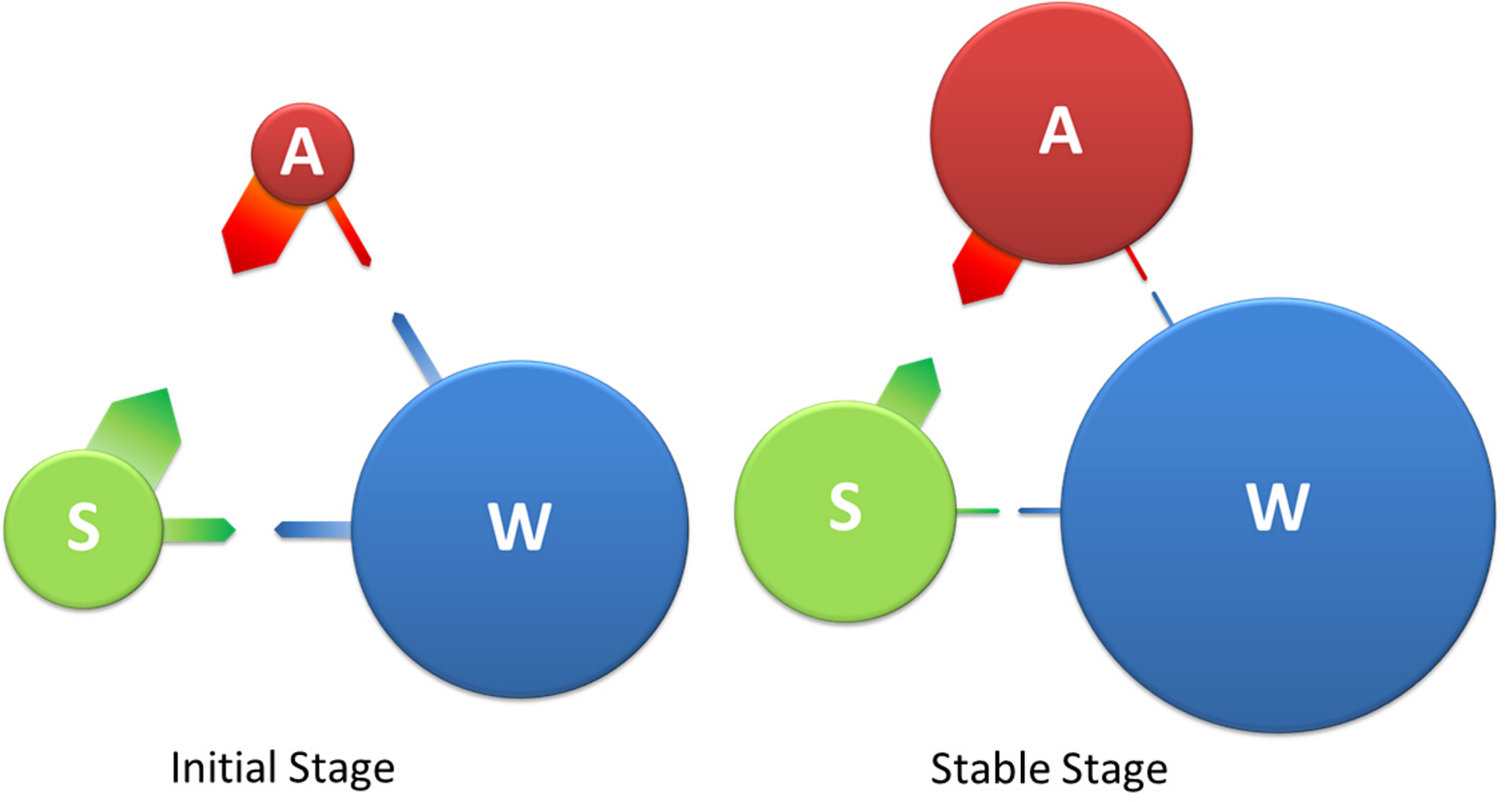
Schematic representation of the microbial interactions in the abalone seed-nursing ecosystem. A, Abalone; S, Adherent substrate; W, Water. The diameters of the circles reflect the inner BC% (BC_An-1—An_%, BC_Sn-1—Sn_%, or BC_Wn-1—Wn_%), and the thicknesses of the arrows reflect the external BC% (for example, the arrow from S to A represents the average value of BC_Sn-1—An_% and BC_Sn—An_%).

To better explain these results, an intuitive image of interactions in the abalone seed-nursing system was generated (Fig 9). This figure illustrates the interactive patterns among different bacterial communities in a system. Diameters of the circles reflect the inner BC% (BC_An-1-An_%, BC_Sn-1-Sn_% or BC_Wn-1—Wn_%). Larger diameters indicate higher stability or inner influence of a community. Thicknesses of the arrows are determined by the external BC% (for example, the arrow from S to A reflects the average value of BC_Sn-1-An_% + BC_Sn-An_%). Thicker arrows indicate a higher external influence of a community. At an early stage in the system, the bacterial community stabilities in abalone, substrate, and water were less (smaller circles) than those in later stages; however, there were more interactions (thicker arrows) between the communities associated with the different components. Over time, the stability of the three microbiota increased (larger circles), exchanges between them decreased (thinner arrows), indicating stabilization of the system.

## Discussion

### Analytical method

The BC% method is based on a method that is widely used for beta diversity studies, namely, the BC similarity index. It is a statistical method used to quantify the compositional similarity between two different communities, based on counts of common species, which correspond to OTUs in this study [19]. BC values are bounded by 0 and 1, where 1 means the two sites have the same composition, and 0 implies no sharing of any species [20]. BC similarity was used in this study because it has two advantages. First, the numbers of OTUs were included in the analysis, which is more accurate than a measure that only considers the presence or absence of OTUs (e.g., the Jaccard index). Secondly, the BC value varies linearly between 0 and 1, so that the simple sum of different BC values can be used to calculate a certain BC% to indicate strength of an influence. Although some other beta diversity indexes are also accurate and widely used (e.g., UniFrac distance), simple sums or percentages cannot be used to show influence. However, different indexes usually highly related. To verify the similarities between different β diversity indexes, BC similarities were compared with weighted UniFrac distances. Results from 149 pairs of data points from the abalone seed-nursing system samples revealed that the two statistical methods were strongly negatively correlated, with an R^2^ of 0.9194 (S6 Fig). As long as an appropriate data conversion is performed, the use of other indexes in a similar analysis may be feasible.

### Effects of bacterial communities on those of the substrate and environment

Li et al. [5] found that the bacterial communities in biofilms varied on different substrata (i.e., glass, acrylic, steel, and hydrophobic glass), and they investigated the effects of bacterial communities on the establishment of plantigrades of the mussel *Mytilus coruscus*. Our results showed that the bacterial communities in abalone affected those of the substrate and water, and especially the substrate. The main reason for this may be the continued accumulation of abalone excrement on the substrate. Interestingly, the developmental stages of abalone appeared to have an effect on the water body (Figs 2 and 3-water). This effect is associated with the transition from differentiation to juvenile abalone stages, but this phenomenon was not observed in the high-throughput sequencing results (Fig 7B). Therefore, further evidence is needed to support this hypothesis. Water provides the living environment and material support for abalone and algae. It must be tolerant and stable to maintain the structure of a system. This study revealed the stability of the bacterial communities in the water. Although the stability of the environment was affected to some extent in early stages of community formation, the BC_W_% value was still high. After external influences declined and bacterial exchanges stabilized, the BC_W_% remained very high (Fig 8). If this balance is interrupted, e.g., water deterioration or pollution [21], the structure of the system might be destroyed. Therefore, the change in the BC% of the environment should be considered a main factor in evaluating system stability.

### Changes in the abalone bacterial community

BC% values describe the spatio-temporal interactions between the components of system. Using the consumer, abalone, as an example, the inner BC_A_% was quite low at the seedling period, indicating that their gut microbiota were unstable. This is similar to observations made in humans and other animals [22]. Development of the intestinal microbiota in infants is also characterized by large rapid changes in microbial composition [10]. Within the first year of life, the enteric microbiota is low in diversity, dominated by only a few bacterial genera and species, but it is also highly dynamic [23,24]. This is likely because a series of barriers exert potent selective pressures on bacteria that arrive in the digestive system during adulthood, but, in the early stages of life, these barriers are low and temporarily allow non-gut-related bacteria to enter the gut [25]. The intestinal microbiota of infants evolve rapidly until they reaches homeostasis at around one year of age. Inter-individual differences then gradually diminish as the microbiota become more complex with age [24], and the microbial population stabilizes and resembles that of an adult [23]. This is similar to the progression in abalone. Because the lifespan of an abalone is shorter than that of a human, the BC_A_% values in this organism increase rapidly early in development (before day 11), which is much quicker than the 1 year required for stabilization in an infant. However, regardless of the length of the unstable period, the gut microbiomes of humans and other animals eventually reach a mature homeostasis. Interestingly, the bacterial population in abalone spat and juvenile (*H*. *diversicolor*) are completely different from those in mature disk abalone (*H*. *discus*) [26]. This big difference may stem from their different growth stages and the geographical habitat.

### Substrate effects on the consumer

The development of abalone can be divided into five stages: the trochophore (0–2 days), creeping larva (2–4 days), peristomial shell larva (4–12 days), differentiation (12–21 days), and juvenile (after 21 days) stages. From our data, the composition of bacterial communities in abalone at different developmental stages were observed to be different, and, from day 0 to day 21, bacterial diversity gradually increased. A study on bacterial diversity in oysters also showed that microbiota associated with the postlarvae stages and adults differed substantially, and that postlarvae stages showed higher bacterial diversity and richness than that in adults of the same species [27]. Because different animals have different diets, food may be one of the main reasons for this difference [28]. At an early stage in development, when the abalone bacterial community was not completely established, and the bacteria in the phycosphere had a strong influence on the consumer, the BC_S-A_% approached 70%. Even at a nearly stable stage, the BC_S-A_% was still high (41.4%). There is increasing evidence that, although a consumer’s gut microbial community can partially determine the nutritional value of food, food ultimately shapes the gut microbiota and metagenome [29]. Milk from healthy mothers, which contains up to 10^9^ microbes/liter from different bacterial groups [30] is a continuous source of bacteria for the infant gut [31]. The presence of identical strains in both the mothers’ breast milk and their infants’ fecal samples suggests an important role for breast milk as a source of early gut colonizers in infants [32,33]. A case study that followed the development of the microbiota in an infant from birth to 2.5 years revealed the strong influence that diet had on changes in the microbial community and the genes responsible for nutritional degradation and synthesis [34]. Favier *et al*. [35] showed that breastfeeding status, weaning, and the successive introduction of different types of table foods all corresponded to rapid shifts in the pattern of bacteria in infant guts. Of note, our PCA results (Fig 7) revealed that changes to the microbiomes of the consumers occurred with changes to the microbiomes of their food (the producers), indicating that the food we eat may instantly change our bacterial communities. However, this effect was only observed inside of the host. With outer characteristics such as growth and health, the effects of changes in food are gradual.

### Environmental effects on the consumer

Lokmer et al. [36] found a high connectivity between seawater communities and the microbiota in the hemolymph of adult oysters. Variation in oyster microbiota is strongly influenced by anatomical site and conditions of the immediate environment [11,27]. Environment may have more influence at an early stage in bacterial community development, before inner stability is achieved. The mode of delivery (vaginally or by cesarean section) of human infants represents the first environment of a newborn. Analysis of the bacterial communities of newborns’ meconiums revealed a strong correlation between the first communities in the digestive tract and microbial communities of either their mothers’ vaginas, in the case of vaginal delivery, or their mothers’ skin, in the case of cesarean section [33,37]. Consequently, samples from the first few days of life often cluster with other very early samples and sometimes with samples from anatomical sites in their mothers, for example, breast milk or vaginas [23]. Although the seemingly chaotic progression in the early gut colonization of newborns was not observed in this study, we observed an obvious decrease in BC_W-A_% and BC_W-S_% between days 7 and 11 (Fig 8). If samples are sequenced during the early days of community establishment (e.g., day 1 or 2), the effects of the environmental microbiome on consumers and producers are likely to be more pronounced than those from either consumers or producers. However, these species may do not rely on particular bacteria because larvae do not reliably encounter the same bacteria in the aquatic habitats that they develop in. The bacterial population that develops in the initial stages of microbiome development is to a significant extent determined by the specific bacteria to which a host happens to be exposed. Over time, the fitness advantages of communities that typically dominate the mature gut environment apparently overcome those of the early-colonizing opportunists that are less well-adapted [23].

## Conclusion

In this study, we identified changes of bacterial communities during seedling cultivation of an herbivorous juvenile aquatic invertebrate animal. We found that the microbiota of abalone at different developmental stages differed significantly, and the community in water might also have been affected to some extent. Further, we spatio-temporally analyzed the microbiota between the plant-feed host and the surrounding water. First, bacterial populations of the consumer and producer were more closely to each other than to that of the environment, as foods can quickly affect the gut microbiota of a host. Second, the spatio-temporal interactions between different ecological components were interpreted by BC% analysis. Consumers and producers showed similar changes in their inner stabilities and responses to external influences. However, the environment, as a nonliving component of the system, had a much more stable inner BC_W_% than either the consumer or producer, demonstrating the importance of environmental stability to an ecosystem. Based on the results of this study, the BC% method was shown to be a good quantitative analytical method. This method can be applied to other ecological studies, such as the evaluation of disease processes in closed ecosystems, optimizing artificial breeding or aquaculture protocols, supporting studies on feed additives or probiotics, and analyzing the effects of invasive species on a system.

## Supporting information

S1 Fig Denaturing gradient gel electrophoresis profiles of all samples in the abalone seed-nursing system. Results of 8% w/v polyacrylamide gels with denaturing gradients that ranged from 55 to 75%, with 1× TAE running buffer and run at 60°C for 11 h at 100 V. Gels were stained with 1× SYBR Gold Nucleic Acid Gel Stain (Life Technologies, Carlsbad, CA, USA) for 30 min.
(TIF)

S2 Fig Unweighted phylogenetic trees showing bacterial in all samples in the abalone seed-nursing system based on their denaturing gradient gel electrophoresis profiles. Trees were constructed using the unweighted pair group method with arithmetic averages (UPGMA).
(TIF)

S3 Fig Sequence length distribution in pyrosequencing data from the abalone seed-nursing system. (TIF)

S4 Fig Rarefaction analysis result of all samples. (TIF)

S5 Fig Relative abundance of major bacterial genera recovered from each sample in the abalone seed-nursing system. (TIF)

S6 Fig Correlations between Bray-Curtis similarity indexes and weighted UniFrac metrics. Plot is based on 149 pairs of data points from the abalone seed-nursing system samples.
(TIF)

S1 Table Statistics of sample reads. (DOCX)
