# Supplementary figures and images for: Relationships between and formation dynamics of the microbiota of consumers, producers, and the environment in an abalone aquatic system
Source: PLoS One. 2017 Aug 7;12(8):e0182590. doi: 10.1371/journal.pone.0182590 (PMC5546691; doi:10.1371/journal.pone.0182590)

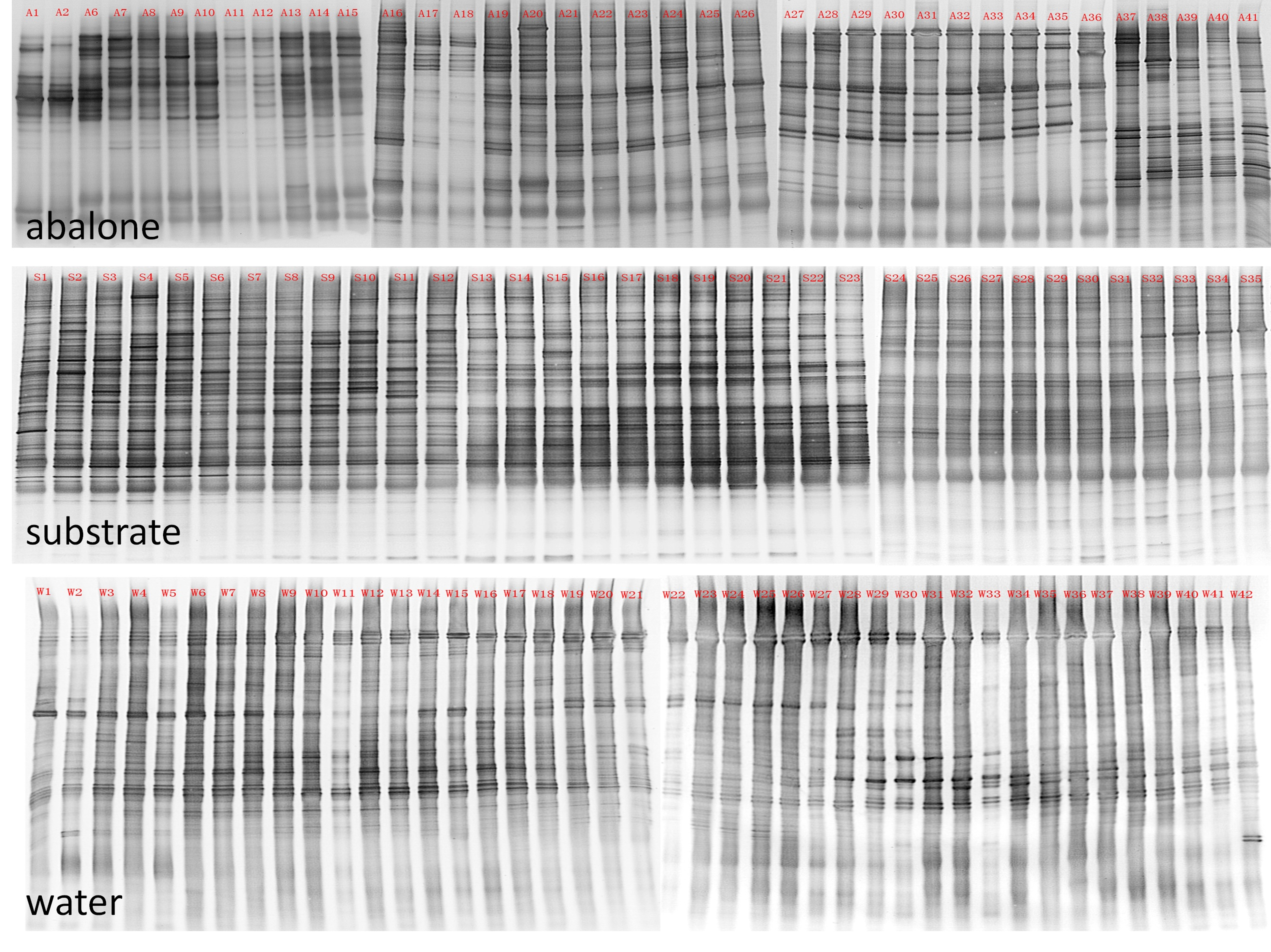

Supplement: S1 Fig — Results of 8% w/v polyacrylamide gels with denaturing gradients that ranged from 55 to 75%, with 1× TAE running buffer and run at 60°C for 11 h at 100 V. Gels were stained with 1× SYBR Gold Nucleic Acid Gel Stain (Life Technologies, Carlsbad, CA, USA) for 30 min. (TIF) [file pone.0182590.s001.tif]

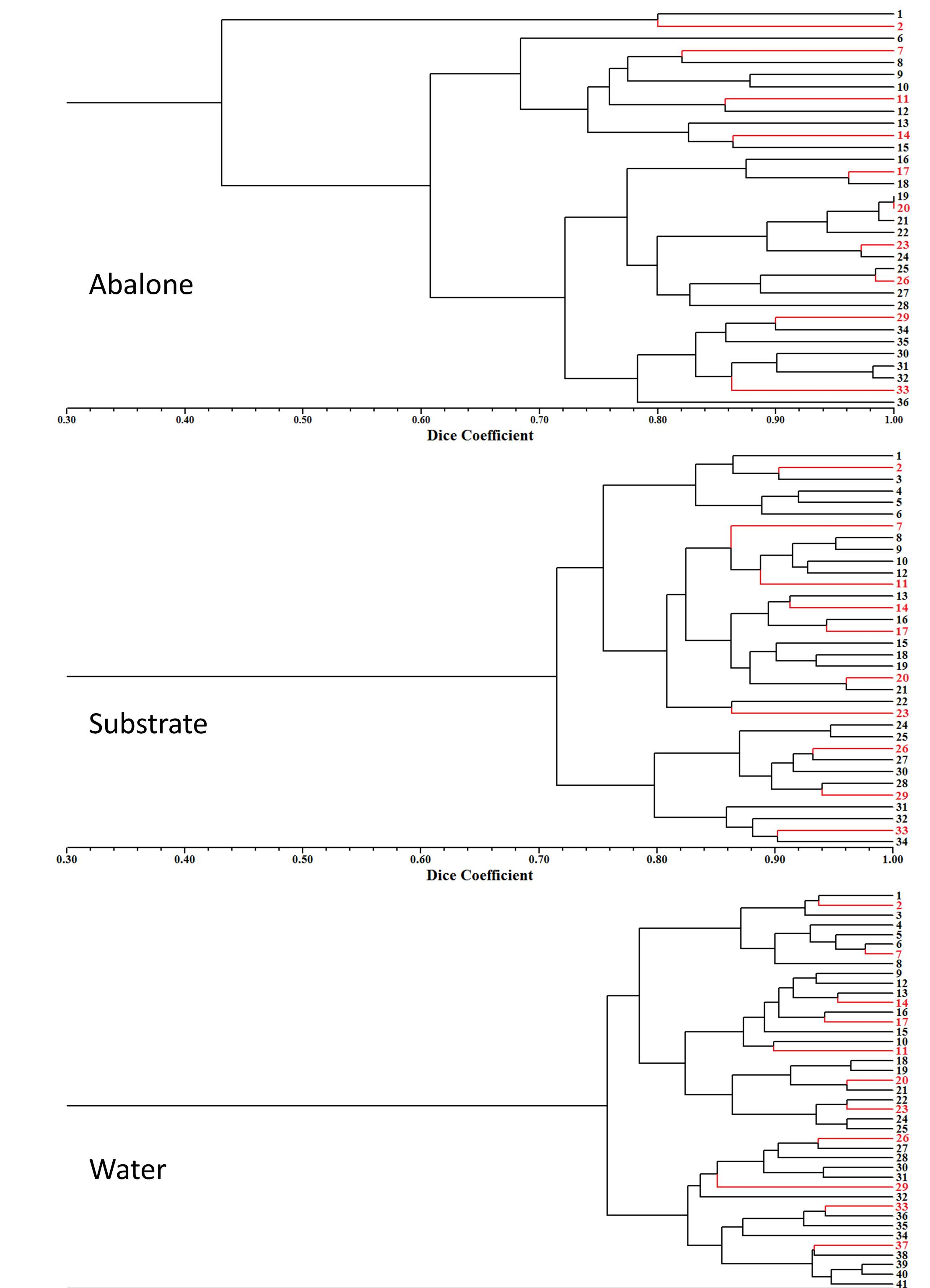

Supplement: S2 Fig — Trees were constructed using the unweighted pair group method with arithmetic averages (UPGMA). (TIF) [file pone.0182590.s002.tif]

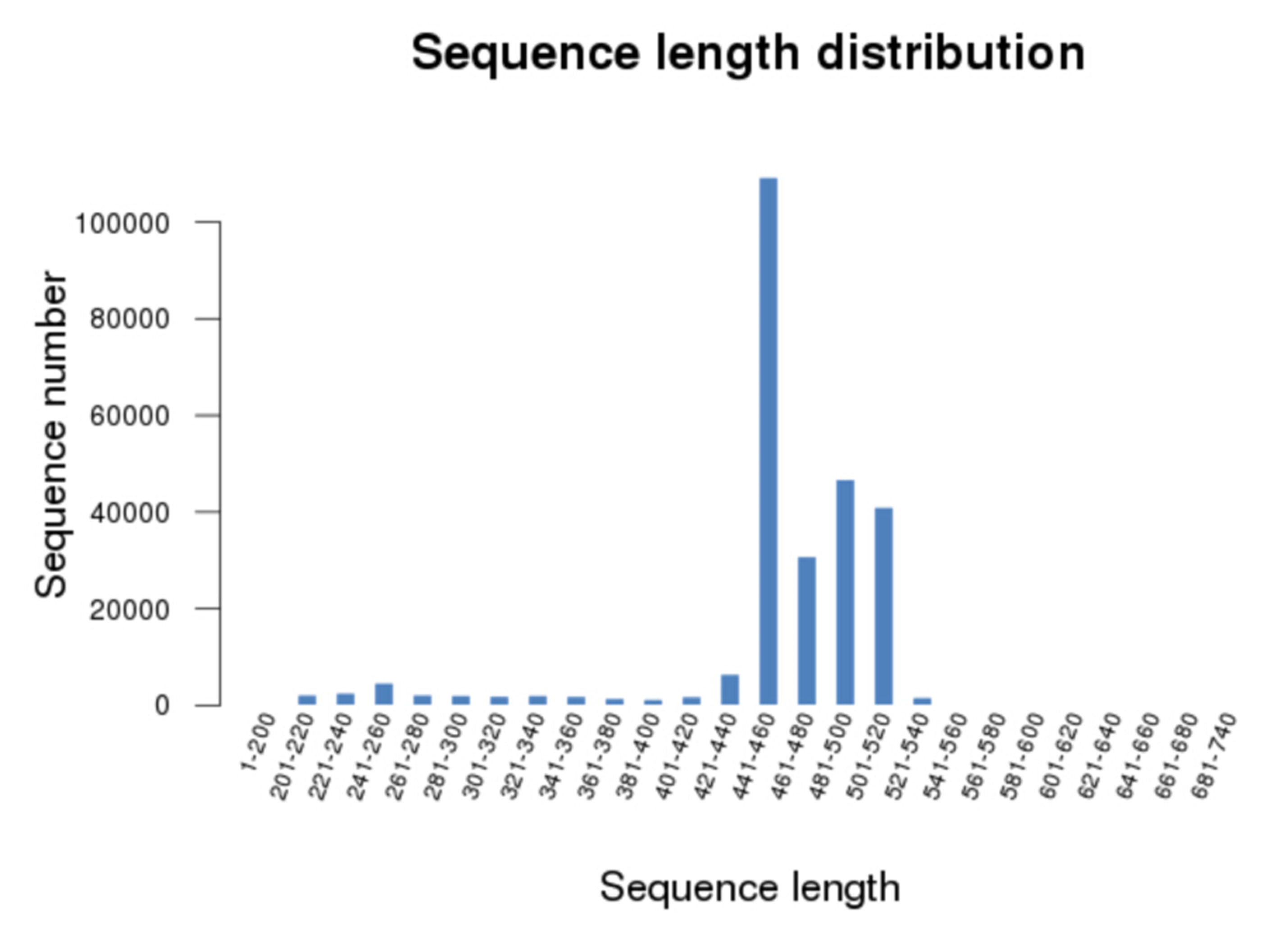

Supplement: S3 Fig — (TIF) [file pone.0182590.s003.tif]

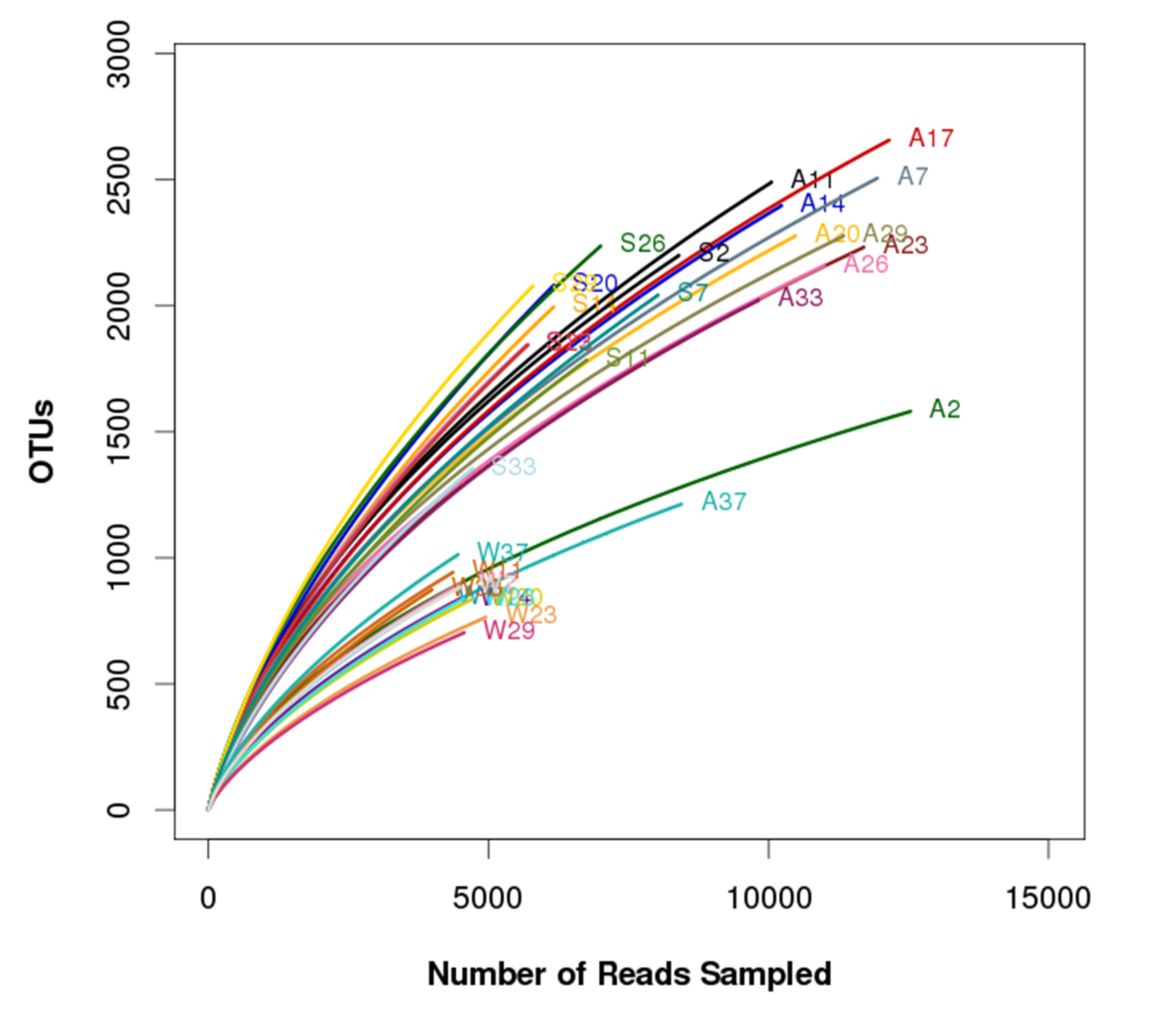

Supplement: S4 Fig — (TIF) [file pone.0182590.s004.TIF]

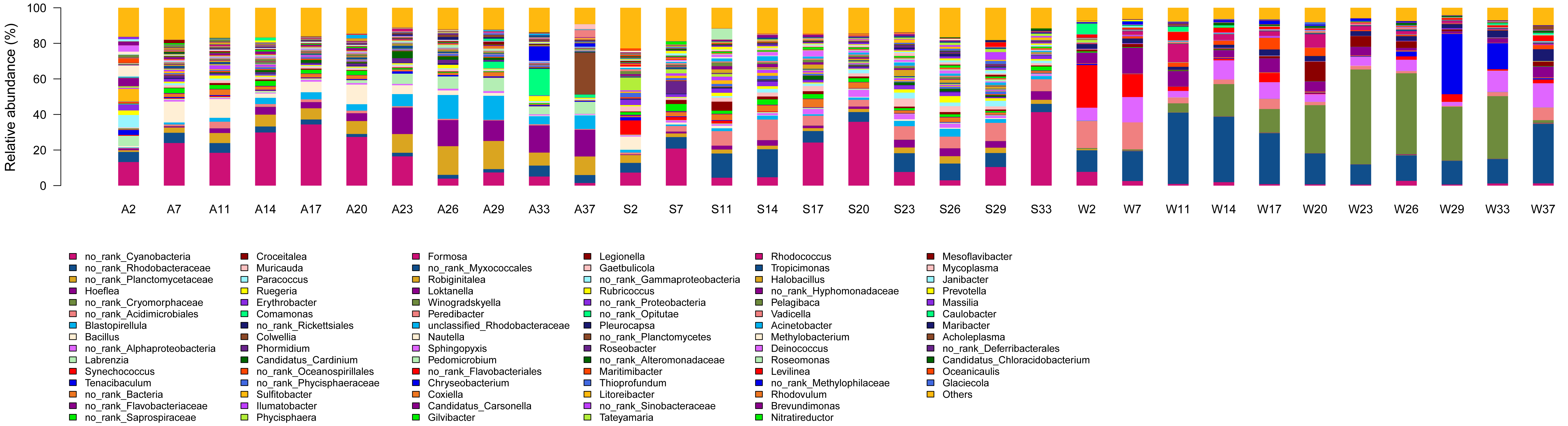

Supplement: S5 Fig — (TIF) [file pone.0182590.s005.tif]

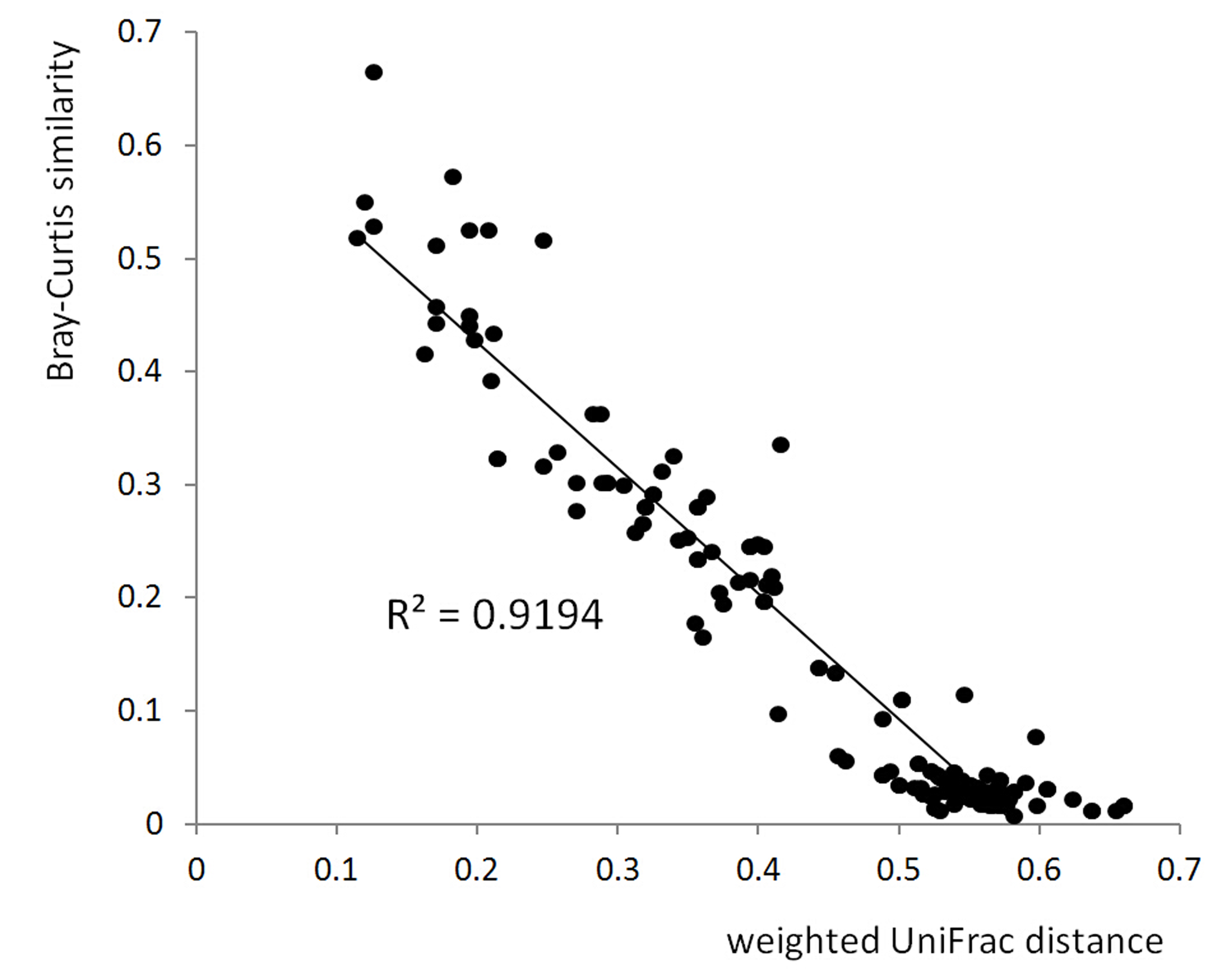

Supplement: S6 Fig — Plot is based on 149 pairs of data points from the abalone seed-nursing system samples. (TIF) [file pone.0182590.s006.tif]
