# Supplementary material for: Relationships between and formation dynamics of the microbiota of consumers, producers, and the environment in an abalone aquatic system
Source: PLoS One. 2017 Aug 7;12(8):e0182590. doi: 10.1371/journal.pone.0182590 (PMC5546691; doi:10.1371/journal.pone.0182590)
Supplement: S1 Table — (DOCX) [file pone.0182590.s007.docx]

Table S1. Statistics of sample reads

| Sample | Valid | Trimmed | Percent |
| --- | --- | --- | --- |
| A2 | 16051 | 14134 | 88.06% |
| A7 | 14555 | 12866 | 88.40% |
| A11 | 12149 | 10764 | 88.60% |
| A14 | 12544 | 11014 | 87.80% |
| A17 | 14727 | 12934 | 87.83% |
| A20 | 12745 | 11180 | 87.72% |
| A23 | 14247 | 12612 | 88.52% |
| A26 | 13481 | 12004 | 89.04% |
| A29 | 13503 | 12137 | 89.88% |
| A33 | 12055 | 10636 | 88.23% |
| A37 | 9961 | 9046 | 90.81% |
| S2 | 12378 | 9237 | 74.62% |
| S7 | 11431 | 9290 | 81.27% |
| S11 | 11604 | 8095 | 69.76% |
| S14 | 11120 | 7892 | 70.97% |
| S17 | 10447 | 6936 | 66.39% |
| S20 | 11108 | 7557 | 68.03% |
| S23 | 10242 | 7318 | 71.45% |
| S26 | 12171 | 8594 | 70.61% |
| S29 | 10347 | 7343 | 70.97% |
| S33 | 7193 | 5669 | 78.81% |
| W2 | 6464 | 5080 | 78.59% |
| W7 | 6537 | 4937 | 75.52% |
| W11 | 7301 | 5142 | 70.43% |
| W14 | 6889 | 4869 | 70.68% |
| W17 | 6603 | 4487 | 67.95% |
| W20 | 6540 | 4883 | 74.66% |
| W23 | 7142 | 5156 | 72.19% |
| W26 | 6179 | 4720 | 76.39% |
| W29 | 6424 | 4814 | 74.94% |
| W33 | 5732 | 4366 | 76.17% |
| W37 | 6598 | 4874 | 73.87% |
